# Long-Term Growth Hormone Associated With High Risk of Acute Kidney Damage

**DOI:** 10.7759/cureus.78680

**Published:** 2025-02-07

**Authors:** Ahmed Akl, Abdullah A Kamal, Zeyad T Olfat, Rowaid Yazbik

**Affiliations:** 1 Nephrology, Dr. Soliman Fakeeh Hospital, Jeddah, SAU; 2 Internal Medicine, Dr. Soliman Fakeeh Hospital, Jeddah, SAU

**Keywords:** acute kidney injury, growth hormone deficiency, podocyte vacuolization, rhgh, somatropin, tubular injury

## Abstract

Growth hormone deficiency (GHD) in children leads to stunted growth and short stature, requiring daily subcutaneous recombinant human growth hormone (rhGH) administration. While rhGH therapy has a well-established efficacy and safety profile, it has been associated with renal complications, including glomerular hyperfiltration, renal hypertrophy, and glomerulosclerosis. However, the association between rhGH and acute kidney injury (AKI) remains unexplored.

We report a 15-year-old male with a known history of GHD managed with daily somatropin therapy. The patient presented with a 4-day history of upper respiratory tract infection, vomiting, diarrhea, and bilateral flank pain after receiving non-steroidal anti-inflammatory drugs (NSAIDs) and antibiotics (cefixime and metronidazole). Initial creatinine levels were elevated at 2.8 mg/dL, rising to 4 mg/dL within days, without evidence of dehydration, proteinuria, or urinary casts. Extensive workup, including autoimmune panels (antineutrophil cytoplasmic antibodies (ANCA), antiphospholipid, complement levels), was unremarkable. A renal biopsy revealed significant vacuolization of tubular and podocyte cells without necrosis or inflammation. rhGH therapy was discontinued and the patient received pulse methylprednisolone therapy (500 mg IV for 3 days) followed by oral prednisolone. Renal function improved and the patient was discharged with stable creatinine and normal kidney function.

This case highlights a potential link between rhGH therapy and AKI, suggesting that growth hormone may exacerbate tubular injury under certain conditions, such as infection and NSAID use. Further research is required to investigate the pathophysiology of rhGH-related kidney injury and identify at-risk populations.

## Introduction

A lack of growth hormone (GH) in youngsters causes stunted growth and, eventually, smaller stature as an adult. Growth hormone must be administered daily by subcutaneous injection as part of the management of GH deficiency, which can be very difficult for young patients [1]. For more than 30 years, recombinant human growth hormone (rhGH) has been used to treat GH deficiency in children, showing significant efficacy and a solid safety record [2]. However, glomerular hyperfiltration, kidney hypertrophy, and glomerulosclerosis can all be brought on by rhGH therapy [3].

On the other hand, acute kidney injury (AKI), which usually lasts no longer than seven days, is defined as a sudden deterioration in renal function that is marked by increased serum creatinine (Cr) and decreased urine output [4]. Classifications of acute kidney injury severity criteria are based on increases in serum creatinine and a decrease in urine volume [5-6]. AKI is consistently linked in research to increased risks of morbidity and mortality [7-8]. The role of rhGH in acute renal failure is not yet studied. Here, we report the case of a child who had acute kidney injury and had been taking daily growth hormone.

## Case presentation

A 15-year-old male child with a history of appendectomy in 2021 and growth hormone deficit was on somatropin. He had previously visited another medical facility with a history of upper respiratory tract infection four days prior, three episodes of vomiting-associated non-bloody diarrhea, and bilateral flank pain for which he was prescribed cefixime, metronidazole, and non-steroidal anti-inflammatory drugs (NSAIDs). His creatinine level was discovered to be 1.8 mg/dl during standard testing. Similar symptoms and an elevated blood creatinine level of 2.8 mg/dl were noted when the patient arrived at our emergency room. The patient's brother had a positive history of ulcerative colitis.

Examination revealed no symptoms of excess fluid or dehydration. Serum creatinine was initially found to be 2.8 mg/dl and urine analysis revealed no cast or white blood cells (WBCs). Electrolytes were within normal limits and venous blood gas first showed respiratory acidosis before improving in repeated venous blood gas. The 24-hour urine protein collection showed 365 mg/day, and over the next two days, serum creatinine was trending up to 4 mg/dL. Antiphospholipid was negative, complements (C3) and (C4) were normal, anti-neutrophilic cytoplasmic autoantibody (ANCA) was normal, and antinuclear antibodies were normal.

**Figure 1:**
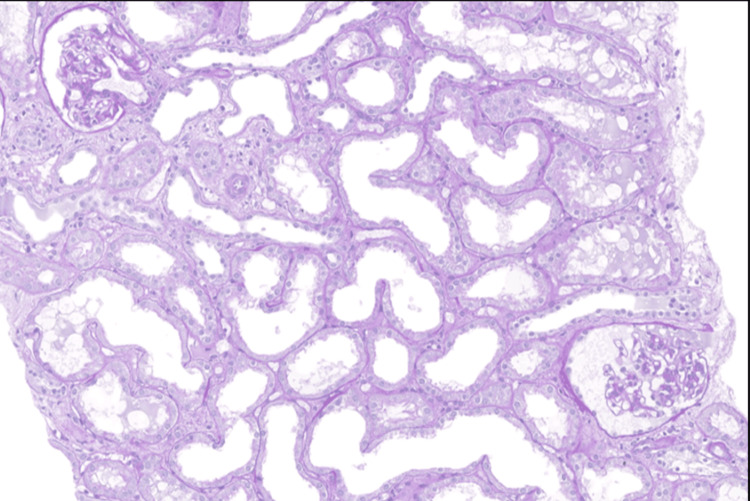
Light microscopy Sections of the kidney biopsy were stained using standard histological protocols, including Hematoxylin and Eosin (H&E), Periodic Acid-Schiff (PAS), Trichrome, and Jones’ Silver stains at 400x magnification (40x objective with 10x eyepiece). Proximal tubules showed extensive loss of brush borders with attenuation and cytoplasmic vacuolation. Occasional uromodulin casts were seen without tubular injury. The glomerular basement membranes did not show thickening, spikes or vacuoles. Adhesions, segmental sclerosis, hyalinosis, fibrin, necrosis or endocapillary proliferation or crescents were not seen. Tubular atrophy and interstitial scarring were not seen. Four interlobular arteries were included and showed no lesion.

**Figure 2:**
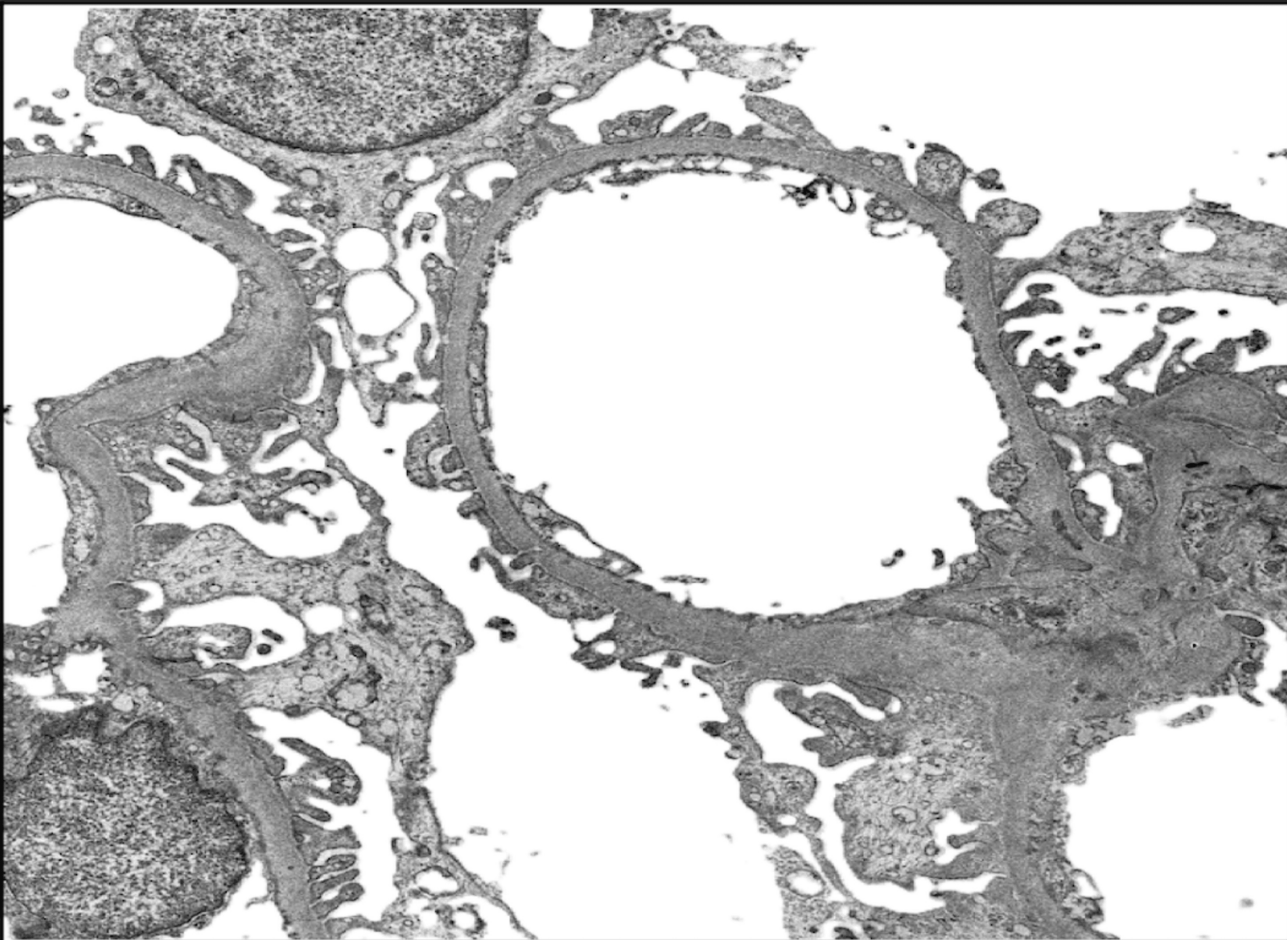
Electron microscopy The podocytes showed vacuolation, and foot processes were generally preserved. There was segmental mild thickening with wrinkling of the glomerular basement membrane and widening of the subendothelial space, observed at an original magnification of 20,000x, without mesangial cell interposition or deposits. The mesangial matrix was mildly increased without deposits. Endothelial cells showed segmental partial loss of fenestrations. The tubular epithelial cells showed attenuation, cytoplasmic vacuolation, and disrupted mitochondria, with brush borders focally lost. No electron-dense deposits were identified.

**Figure 3:**
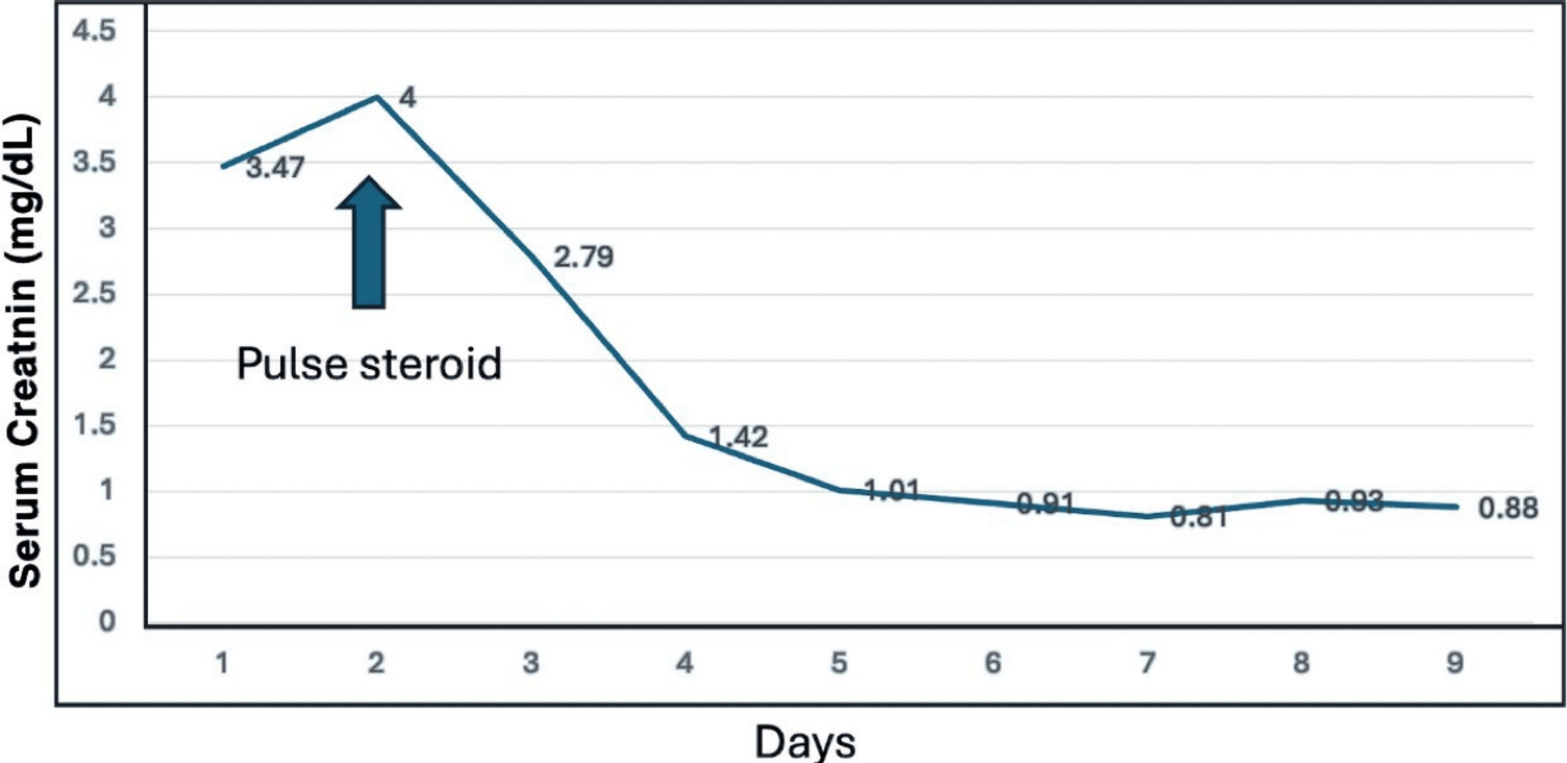
Monitoring of serum creatinine during the course of management The trend shows dramatic improvement in serum creatinine after starting pulse steroid.

Growth hormone was stopped. Renal biopsy was done, which revealed aggressive vacuolization of the podocytes and tubular cells with no necrosis nor cellular infiltrations and immunofluorescence: sections that contained 5 perfused glomeruli were stained for C1q, C3, IgG, IgM, IgA, and Kappa and Lambda light chains; all immunostains were negative (Figures 1, 2). Also, the patient was suspected to have celiac disease; laboratory work-up and colonic biopsy were normal. Once the patient received pulse methylprednisolone 500 mg IV for 3 days, serum creatinine started to improve and the patient was discharged on oral prednisolone 20 mg once daily with follow-up in the nephrology clinic (Figure 3). Currently, the patient is enjoying normal kidney function with no proteinuria and is off steroids or any medication.

## Discussion

Current studies on the use of growth hormone by athletes for muscle mass gain indicate potential effects on kidney function through various mechanisms, both direct and indirect, including alterations in kidney size, glomerular filtration rate (GFR), and tubular function. However, these effects remain unclear and require further research [9]. In certain cases, such as renal transplant patients receiving rhGH, final adult height has been shown to improve with rhGH treatment [10]. However, in our case, the patient had been taking nonsteroidal anti-inflammatory drugs (NSAIDs) for pain before admission, which could have contributed to acute kidney injury. Nevertheless, the patient did not receive a dose high enough to significantly impact kidney function and did not develop interstitial nephritis** **[11].** **

Because his brother had ulcerative colitis, the patient was determined to be at high risk for autoimmune rapid progressive glomerulonephritis. However, both the autoimmune screen and the patient's colonic biopsies came back negative for ulcerative colitis. It has been discovered that growth hormone directly increases podocyte permeability to proteins. This is because GH causes podocyte damage by downregulating E- and P-cadherins. GH causes cultured podocytes to express more transforming growth factor-beta-induced protein (TGFBIp), and the cell supernatant exhibits progressively higher TGFBIp secretion [12]. 

In a study by D'Ercole and associates, at various intervals following growth hormone administration, the researchers assessed the organ concentrations of insulin-like growth factor I (IGF-I) in rats. Compared to plasma, IGF-I concentrations increased in the liver and kidneys earlier and more quickly. This raises the question of whether blood-borne IGF-I may impact renal function under normal circumstances or if it simply reflects the rate of IGF-I synthesis in these organs. It also shows that plasma IGF-I may be obtained, at least in part, from IGF-I created by these organs [13]. These findings can help to explain the features of our patient's renal biopsy, which showed extensive podocyte and tubular vacuolization together with the loss of the tubules' brush border. While growth hormone level was normal at presentation.

The patient responded appropriately to the steroid once it was started, and the kidney biopsy revealed that the patient did not meet the rapidly progressive glomerulonephritis​​​​​​​ (RPGN)and had a negative autoimmune panel. Following steroid initiation and follow-up, the patient's creatinine and follow-up urine protein levels returned to normal. Steroids are functional GH antagonists because they influence both the release and effects of GH at the target sites [14].

In conclusion, a patient's acute kidney injury may be caused by a variety of factors, but the patient's lengthy history of growth hormone use, severe podocytopathy, and quick recovery following growth hormone cessation and a brief course of steroids suggest an increased likelihood of growth hormone-induced glomerular injury.

## Conclusions

This case highlights a potential link between rhGH therapy and AKI, suggesting that growth hormone may exacerbate tubular injury under certain conditions, such as infection and NSAID use. Further research is required to investigate the pathophysiology of rhGH-related kidney injury and identify at-risk populations.
